# *Escherichia coli* Nissle 1917 Antagonizes *Candida albicans* Growth and Protects Intestinal Cells from *C. albicans*-Mediated Damage

**DOI:** 10.3390/microorganisms11081929

**Published:** 2023-07-28

**Authors:** Yasmine Rebai, Lysett Wagner, Mayssa Gnaien, Merle L. Hammer, Mario Kapitan, Maria Joanna Niemiec, Wael Mami, Amor Mosbah, Erij Messadi, Helmi Mardassi, Slavena Vylkova, Ilse D. Jacobsen, Sadri Znaidi

**Affiliations:** 1 Laboratoire de Microbiologie Moléculaire, Vaccinologie et Développement Biotechnologique (LR16IPT01), Institut Pasteur de Tunis, University of Tunis El Manar, Tunis 1068, Tunisia; yassmine.rebai@gmail.com (Y.R.);; 2 Septomics Research Center, Friedrich Schiller University, 07745 Jena, Germany; 3 Leibniz Institute for Natural Product Research and Infection Biology—Hans Knöll Institute, 07745 Jena, Germany; 4 Center for Sepsis Control and Care, 07747 Jena, Germany; 5 Plateforme de Physiologie et Physiopathologie Cardiovasculaires (P2C), Laboratoire des Biomolécules, Venins et Applications Théranostiques (LR20IPT01), Institut Pasteur de Tunis, Université Tunis El Manar, Tunis 1068, Tunisia; 6 Laboratory of Biotechnology and Bio-Geo Resources Valorization (LR11ES31), Higher Institute of Biotechnology of Sidi Thabet (ISBST), University of Manouba, Tunis 2010, Tunisia; 7 Institute of Microbiology, Friedrich Schiller University, 07743 Jena, Germany; 8 Institut Pasteur, Institut National de la Recherche Agronomique (INRA), Département Mycologie, Unité Biologie et Pathogénicité Fongiques, 75015 Paris, France

**Keywords:** antifungal, *Escherichia coli*, Nissle 1917, probiotics, *Candida albicans*, polymicrobial interactions

## Abstract

*Candida albicans* is a pathobiont of the gastrointestinal tract. It can contribute to the diversity of the gut microbiome without causing harmful effects. When the immune system is compromised, *C. albicans* can damage intestinal cells and cause invasive disease. We hypothesize that a therapeutic approach against *C. albicans* infections can rely on the antimicrobial properties of probiotic bacteria. We investigated the impact of the probiotic strain *Escherichia coli* Nissle 1917 (EcN) on *C. albicans* growth and its ability to cause damage to intestinal cells. In co-culture kinetic assays, *C. albicans* abundance gradually decreased over time compared with *C. albicans* abundance in the absence of EcN. Quantification of *C. albicans* survival suggests that EcN exerts a fungicidal activity. Cell-free supernatants (CFS) collected from *C. albicans*-EcN co-culture mildly altered *C. albicans* growth, suggesting the involvement of an EcN-released compound. Using a model of co-culture in the presence of human intestinal epithelial cells, we further show that EcN prevents *C. albicans* from damaging enterocytes both distantly and through direct contact. Consistently, both *C. albicans*’s filamentous growth and microcolony formation were altered by EcN. Taken together, our study proposes that probiotic-strain EcN can be exploited for future therapeutic approaches against *C. albicans* infections.

## 1. Introduction

The compositional and functional alteration of the gut microbiome—also termed dysbiosis—is a pathological condition driven by a set of environmental and host-related factors that perturb the microbial ecosystem [1]. A dysbiotic state is observed in the context of many diseases and health conditions, including inflammation of the gut (e.g., inflammatory bowel disease), obesity and cancer, but also during immunosuppression (e.g., in patients receiving organ transplants). It can also be directly induced through the administration of large-spectrum antibiotics, leading to a dramatic decline in bacterial diversity [2]. Because the gastrointestinal microbiome includes viral [3], bacterial [4] and fungal (also termed mycobiome) microbiota, a dysbiotic state can translate into an expansion of intestinal fungal pathobionts, particularly *Candida albicans* (*Ca*), a fungus that can grow out of proportion to cause disease or participate in disease onset/severity [5,6]. *Ca* is an opportunistic yeast that is also part of the oral and vaginal microbiota. It can cause diverse pathologies in both immunocompetent and immunocompromised individuals, ranging from oral or vaginal candidiasis (i.e., superficial infections) to life-threatening systemic candidiasis (i.e., invasive infection among severely immunocompromised patients), where translocation across the digestive intestinal barrier is achieved by *Ca* [7,8]. This process is dynamic, initiated by invasion and followed by cellular damage and loss of epithelial integrity [9]. The process occurs following a morphological change of the fungus, called yeast-to-hypha transition, which facilitates tissue invasion and mucosal damage [10]. Alternatively, *Ca* can first form microcolonies on the epithelium and then invade tissues from the colony [11].

Numerous therapeutic strategies are currently under development to deplete the opportunistic fungi or to enhance the beneficial microbiota. One of the novel treatment therapies for dysbiosis includes fecal microbiota transplantation, i.e., supplementation with healthy gut microbiota. However, concomitant *Candida* overgrowth in recipients with certain disease conditions (e.g., infection with *Clostridium difficile*) or a high abundance of *Ca* in donor stool correlate with reduced efficacy of the treatment [12]. Alternatively, treatment with antifungal agents appears to, rather, increase the severity of the underlying disease (e.g., colitis) and exacerbate the development of allergic airway disease [13]. It also leads to a relative increase in several specific fungal species that are present at low levels, such as *Aspergillus amstelodami*, *Epicoccum nigrum* and *Wallemia sebi* [13]. Consequently, a more successful treatment could rely on the use of live bacteria with probiotic properties. They can reverse intestinal fungal overgrowth owing to potent antimicrobial activities, and contribute to restoring microbial balance in the gut.

Several studies have focused on the antimicrobial potential of probiotic bacteria, particularly via the natural secretion of metabolites with antifungal properties [14]. These bacteria lead to prospects for their use as therapeutic agents. In this context, a probiotic bacterial strain called *Escherichia coli* Nissle 1917 (EcN) is probably the most intensively investigated bacterial strain and has been industrially produced and marketed since 1971 as the licensed pharmaceutical product Mutaflor™ [15]. It was isolated in 1917 by Alfred Nissle from the stool of a World War I soldier, who was the only one of his unit not suffering from dysentery. EcN is capable of modifying the intestinal microflora through its antimicrobial and anti-inflammatory activities. For example, EcN produces microcins mediating direct antagonistic activity against enteropathogenic bacteria [16]. EcN also reduces the survival of antibiotic-treated bacterial persisters by antagonizing the drug-treated cells in a contact-dependent manner [17]. Cell-free supernatants from EcN inhibited growth of antimicrobial-resistant *Salmonella Typhimurium*, *E. coli* and *Klebsiella oxytoca* [18]. In addition, EcN is indirectly antagonistic to enteropathogens through signaling to the host intestinal epithelium [19]. However, it is still unknown whether EcN exhibits a direct antagonistic effect against *Ca*, and how efficient it could be at preventing *Ca* from damaging intestinal cells and causing disease.

There exists a complex relationship between *Ca* and gut-associated bacteria. For instance, *Enterococcus faecalis* can promote the commensal state of *Ca* via the secretion of bacterial products that alter cellular morphology and inhibit biofilm formation [20]. Some *E. coli* strains, such as MG1655, can directly inhibit growth or kill *Ca* through the release of fungicidal factors [21]. Bacteria belonging to different genera, such as *Bifidobacterium*, *Streptococcus* and, more extensively, *Lactobacillus*, have been studied with regard to their antagonistic effects against *Ca* [22]. *Lactobacillus rhamnosus* in particular can antagonize *Ca* by reshaping the metabolic environment, forcing metabolic adaptations that reduce *Ca* pathogenicity [23]. On the other hand, certain fungal–bacterial combinations—for example, *C. albicans* and *Proteus mirabilis*—have the potential to result in enhanced virulence and might, therefore, promote *Ca* translocation and dissemination from the gastrointestinal tract [24]. In this report, we investigated the interaction between *Ca* and probiotic strain EcN. We show that EcN antagonizes *Ca* growth and protects intestinal cells from *Ca*-mediated damage, reflecting a strategy to further explore for future therapeutics.

## 2. Materials and Methods

### 2.1. Strains and Growth Conditions

EcN strain used in this study was provided by Ardeypharm Company of Germany. *Ca* reference strain SC5314 and EcN strain were frozen in glycerol stock at −80 °C. Before starting each experiment, strain SC5314 was plated on Yeast Peptone Dextrose (YPD) agar plates (1% yeast extract, 2% peptone, 2% glucose, 2% agar) at 30 °C for 24 h. EcN strain was sub-cultured on Luria Broth (LB) agar plates (1% tryptone, 0.5% yeast extract, 1% NaCl, 2% agar) at 37 °C for 24 h.

### 2.2. In Vitro Co-Culture Experiments

*Ca* SC5314 and EcN strains were co-cultured in YPD rich medium, as previously reported [21]. Overnight, *Ca* cells were diluted in 25 mL of YPD to an OD_600 nm_ of 0.07 and were grown to an OD of 0.2. Overnight, EcN cells were diluted to 1:200 and grown to an OD of 0.2. Once the desired OD was reached, a subset of EcN culture was heat-inactivated by incubating the cells at 65 °C for 2 h. Then, *Ca* SC5314, live EcN and heat-inactivated EcN were diluted in 25 mL of YPD medium to ODs_600 nm_ of 0.1 (*Ca*) and 0.001 (EcN or heat-inactivated EcN), both equivalent to 10^6^ cells. Co-cultures were incubated at 37 °C under vigorous shaking for 18 h. The abundance of the strains as a function of time (in colony-forming units per mL, CFU/mL) was quantified every 2 to 4 h by spreading the co-culture on solid YPD media supplemented with 100 μg/mL of ampicillin (which inhibits the growth of EcN and selects for *Ca* growth) and LB supplemented with 0.5 μg/mL of Amphotericin B (which inhibits the growth of *Ca* and selects for EcN growth). As a control experiment, monocultures of *Ca* strains SC5314 and EcN were also followed over time, and their abundances were determined similarly. The assay was performed 5 times independently.

### 2.3. Growth Curve Assays

Growth assays were performed in 96-well plates (TPP tissue culture plate, polystyrene Z707902, Merck, Darmstadt, Germany) using different media: YPD, Roswell Park Memorial Institute (RPMI) 1640 medium, Dulbecco’s Modified Eagle Medium (DMEM, Gibco, Germany) and LB. EcN and *Ca* overnight cultures were centrifuged (4000× *g*, 10 min) and washed twice with phosphate-buffered saline (PBS) pH 7.4. After OD_600 nm_ measurements, bacterial and fungal cells were adjusted to ODs 2 and 4 in 1 mL PBS, respectively. Ten microliters of each strain was added to 190 µL of the respective medium. The plates were incubated for 24 h in a Tecan spark multimode microplate reader at 37 °C for EcN and at 30 °C for *C. albicans*. The absorbance at 600 nm was measured every 20 min.

### 2.4. Collection of Cell-Free Supernatant (CFS)

The co-culture experiment was performed in YPD as described above. After 8 h of incubation at 37 °C, cultures were centrifuged (4000× *g*, 15 min), then the supernatants were collected followed by filtration using 0.2 µm pore-sized filters. CFSs were buffered with PBS (pH 7.4) and used as growth medium for *Ca* as described above (Section 2.3). CFSs were supplemented with 2% Glucose (D (+) -Glucose, Carl Roth) to correct for glucose consumption. CFS samples from EcN monoculture and EcN-*Ca* co-culture were further subjected to ultrafiltration through Amicon Ultra-15 centrifugal filter devices as per the manufacturer’s instructions (Millipore, Germany), using Amicon 3K (Catalog # UFC900324) and Amicon 10K (Catalog # UFC901024) devices. Doubling times were calculated by converting Tecan OD_600 nm_ readings into flask OD_600 nm_ readings using the following formula: OD_Flask_ = OD_Tecan_ × 12.2716–1.0543 within the exponential *Ca* growth phase as described previously [25].

### 2.5. Intestinal Epithelial Cell Model

The human colonic adenocarcinoma cell line C2BBe1 (CRL-2102™, ATCC; RRID:CVCL_1096) and mucus-secreting HT29-MTX cells (HT29-MTXE12, Sigma, Roedermark, Germany; RRID: CVCL_G356) were cultivated in high-glucose DMEM (Gibco) supplemented with 10% heat-inactivated fetal calf serum (FCS), 0.01 mg/mL holo-transferrin (Merck) and 1% non-essential amino acids (Gibco) and maintained at 37 °C, 5% CO_2_ [24]. The cells were washed with PBS pH 7.4 and then detached via accutase digestion for C2BBe1 and trypsin for HT29-MTX for 10 min at 37 °C. The cell number was determined using a Neubauer chamber by counting a 1:2 mixture of cells with trypan blue, which stains only dead cells. To obtain an infection model for the human gut epithelium, C2BBe1 and HT29-MTX were mixed in a 70%/30% ratio, respectively, to 10^5^ cells/mL in supplemented DMEM and seeded into collagenated polystyrene multi-well plates pretreated with 10 µg/mL collagen I for 2 h at room temperature. The seeded cells matured for 14 days with medium exchange twice per week. Prior to infection, the cells were washed with PBS pH 7.4 and DMEM medium was replaced with Keratinocyte Basal Medium Gold (KBM™ Gold, Lonza, Cologne, Germany).

### 2.6. Intestinal Epithelial Cell Infection Model

After cell seeding and maturation as described above, DMEM medium was replaced with a highly buffered KBM Gold medium prior to the addition of *Ca* and EcN suspensions. For fungal and bacterial infection solution preparation, overnight cultures of *Ca* and EcN were washed twice and harvested (1 min, 10,000× *g*), then resuspended in PBS pH 7.4. Ten microliters of fungal suspension was adjusted to 2 × 10^7^ cells/mL to infect the intestinal epithelial cells at a multiplicity of infection (MOI) of 10. Ten microliters of the bacterial suspension were adjusted to 2 × 10^8^ cells/mL and 2 × 10^9^ cells/mL to infect intestinal cells at ratios of 10:1 (10×) and 100:1 (100×) relative to *Ca* (i.e., bacterial/fungal ratios of 10× and 100×). Uninfected control with PBS instead was always added. The assay was performed in 96-well plates with 200 µL final volume. In this enterocyte infection model, two conditions were tested. In the first condition, intestinal cells were infected first with *Ca*, then 6 h later with EcN (therapeutic model). In the second condition, the intestinal cells were infected first with EcN, then 6 h later with *Ca* (prophylactic model). The assay was performed 5 to 7 times independently under standard cell culture conditions for 24 h at 37 °C with 5% CO_2_.

### 2.7. Cytotoxicity Assay LDH

To measure intestinal cell damage, the supernatant was removed after 24 h of total incubation. The released lactate dehydrogenase from damaged cells was quantified using a colorimetric Cytotoxicity Detection Kit (LDH) according to the manufacturer’s guidelines (Roche, Germany). A high-damage control was used as reference by adding 0.25% (*v*/*v*.) Triton-X-100 to cells. Uninfected cells were used as low-damage control. The absorption at 490 nm (A490nm) was measured and corrected by subtracting the absorption values from the reference wavelength at 660 nm (A660nm). Host cell damage was calculated as follows: the values of uninfected control (PBS) were subtracted as background from all other measurements. Then, the relative intestinal cell damage for each sample was calculated as % Triton-induced LDH release as follows:

$$\frac{A(\mathrm{infection})\;-\;A(\mathrm{low}\;\mathrm{control}\;\mathrm{PBS})}{A(\mathrm{high}\;\mathrm{control})\;-\;A(\mathrm{low}\;\mathrm{control}\;\mathrm{PBS})}\times 100\;$$

### 2.8. Cell Contact Assay

EcN and *Ca* strain SC5314 were separated using 0.4 µm pore-sized hanging PET cell culture inserts (Merck, # MCHT24H48). The assay was performed in 24-well plates (TPP tissue culture plate, polystyrene Z707805, Merck), where 50 µL of bacterial and fungal suspension was used in 1 mL final volume. EcN infection solution was added either into the well in direct contact with *Ca* and intestinal cells or separately into the insert (see experimental design). Incubation and intestinal cell damage quantification were performed as described above. The assay was carried out 3 to 4 times independently.

### 2.9. Microcolony Formation

Microcolony assay was performed as described previously [26], with minor modifications. *Ca* and EcN overnight cultures were washed twice with PBS (1 min, 10,000× *g*). Fungal cells were diluted to 10^3^ cells per well. Bacterial cells were diluted to 10^4^ and 10^5^ to a final concentration 10× and 100× relative to *Ca* cells. The assay was performed in 24-well plates (TPP tissue culture plate, polystyrene Z707805, Merck) in RPMI, then incubated at 37 °C, 5% CO_2_ for 24 h. Microcolonies were observed using a Zeiss Axio Vert A1 microscope. The images were analyzed using ImageJ Fiji software v 2.10. The assay was performed 3 times independently.

### 2.10. Filamentation Assay

*Ca* and EcN overnight cultures were washed twice with PBS pH 7.4 (1 min, 10,000× *g*). Fungal cells were diluted to 10^6^ cells per well. Bacterial cells were diluted to 10^7^ and 10^8^ to a final concentration 10× and 100× relative to *Ca* cells. The assay was performed in 24-well plates (TPP tissue culture plate, polystyrene Z707805, Merck) in RPMI, then incubated at 37 °C, 5% CO_2_. *Ca* hyphae were imaged using Zeiss Axio Vert A1 microscope after 8 h of incubation. The assay was performed 3 times independently.

### 2.11. Statistical Analysis

Data were processed using GraphPad Prism v8 software. Data were statistically analyzed using an unpaired, two-tailed Student *t* test, non-parametric two-tailed Mann–Whitney’s test or Analysis of Variance (ANOVA). *p*-values were subsequently corrected for multiple comparisons using the Bonferroni method. *p*-value statistical significance is indicated as follows: *, *p* < 0.05; **, *p* < 0.01; ***, *p* < 0.001; ****, *p* < 0.0001.

## 3. Results

**Figure 1:**
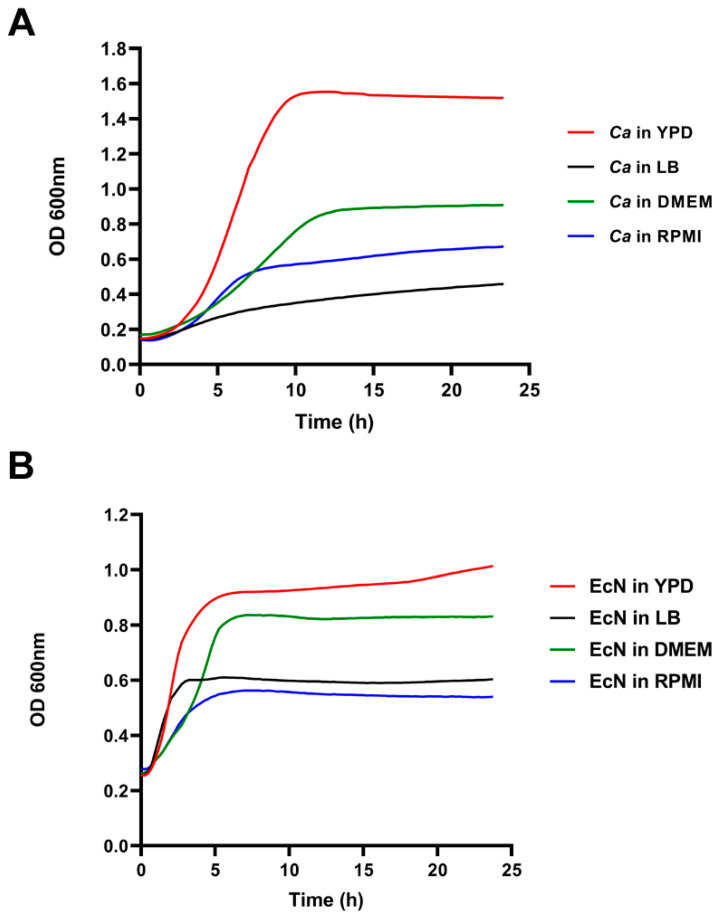
*C. albicans* SC5314 and EcN growth curves in different growth media. (**A**) *Ca* growth (OD_600 nm_, *y*-axis) was measured every 20 min as a function of time (*x*-axis, hours) in LB (black line), YPD (red line), DMEM (green line) and RPMI (blue line) media. A representative growth curve is displayed out of 12 replicates. (**B**) EcN growth (OD_600 nm_, *y*-axis) was measured every 15 min as a function of time (*x*-axis, hours) in LB (black line), YPD (red line), DMEM (green line) and RPMI (blue line) media. A representative growth curve is displayed out of 24 replicates.

*EcN and C. albicans growth characteristics in different media*. Before testing the impact of EcN on *Ca* growth, we sought to determine the optimal growth medium that allows both species to grow efficiently in vitro. We tested four growth media, two of which are standard rich media for *Ca* and *E. coli*—YPD and LB, respectively—and two additional media, RPMI 1640 and DMEM, which are used for mammalian cell culture as well as for testing *Ca’s* ability to filament (See Section 2). *Ca* was able to grow efficiently in YPD (Figure 1A, red curve), less efficiently in RPMI and DMEM media (Figure 1A, blue and green curves, respectively) and poorly in LB medium (Figure 1A, black curve). Poor growth of *Ca* in LB was due to the lack of glucose in this medium, whereas the apparent moderate growth rate of *Ca* in RPMI and DMEM could be due to the activation of filamentous growth, a morphological transition that interferes with OD_600 nm_ readings and could, therefore, translate into an altered growth curve (Figure 1A). EcN, on the other hand, grew more efficiently in YPD and DMEM media (Figure 1A, red and green curves, respectively) than in LB and RPMI media (Figure 1A, black and blue curves, respectively). Because both *Ca* and EcN were able to grow efficiently in YPD, subsequent co-culture experiments were performed in YPD.

**Figure 2:**
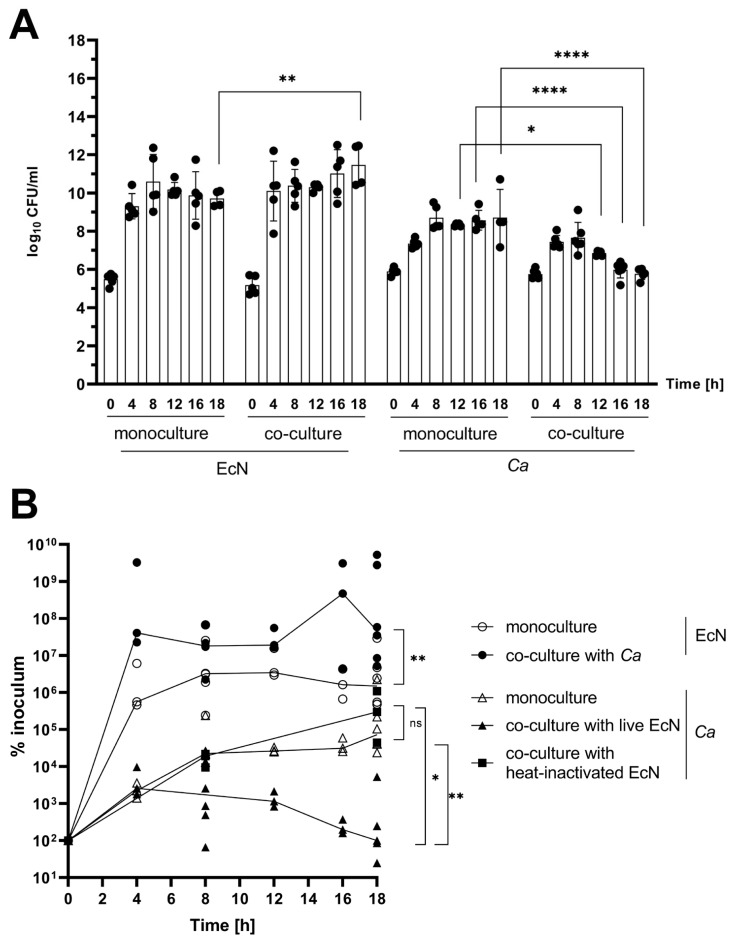
Inhibition of *C. albicans* SC5314 growth by EcN in an in vitro co-culture assay. (**A**) The abundance of each species as a function of time (hours, *x*-axis) in monoculture and co-culture experiments was determined by counting colony forming units per milliliter (CFU) on selective solid agar media. The abundance of each species is expressed as log_10_ CFU/mL (*y*-axis). Data shown on the *y*-axis represent the mean values ± standard deviation from *n* = 5 independent experiments (i.e., performed on different days). Statistical significance was calculated using a two-way ANOVA. *P*-values were subsequently corrected for multiple comparisons using the Bonferroni method (*, *p* < 0.05; **, *p* < 0.01; ****, *p* < 0.0001). (**B**) Percent abundance of EcN grown in monoculture (open circles) or in co-culture with *Ca* (filled circles) and *Ca* grown in monoculture (open triangle) or in co-culture with live (filled triangles) or heat-inactivated (filled squares) EcN relative to the corresponding inoculum (% inoculum, *y*-axis, i.e., survival) as a function of time (hours, *x*-axis) were determined by dividing the corresponding CFU/mL values at each time point (0 h, 4 h, 8 h, 12 h, 16 h and 18 h) by the CFU/mL value at time point 0. Statistical analyses were carried out using a two-tailed Mann–Whitney test comparing monoculture and co-culture % inoculum values for each species at time point 18 h (ns, not significant; *, *p* < 0.05; **, *p* < 0.01).

*EcN inhibits C. albicans growth in a competitive growth assay.* In order to test whether EcN exerts an inhibitory effect on growth of *Ca* when both species are cultivated simultaneously in vitro, we co-cultured *Ca* strain SC5314 with EcN in YPD medium (Figure 2A). We found that the abundance of *Ca* co-cultured with EcN gradually decreased over time, while its growth in monoculture increased over time (Figure 2A, *Ca* histograms compare co-culture to monoculture). On the other hand, the abundance of EcN appeared to further increase when it was simultaneously grown with *Ca*, compared with its abundance during growth in monoculture (Figure 2A, compare EcN monoculture to co-culture at time point 18 h). These results suggest that EcN exerts a growth-inhibitory activity on *Ca* when it is competitively grown with *Ca*.

Next, we tested whether EcN-mediated growth inhibition was fungistatic or fungicidal in nature, and whether it required metabolically active EcN cells. We quantified the percent survival of *Ca* in monoculture and in co-culture with live EcN or with heat-inactivated EcN cells (Figure 2B, see Section 2). We found that the number of viable *Ca* cells decreased over time when co-cultured with live EcN, starting at time point 8 h (Figure 2B, compare curve with open triangles to that with filled triangles). Conversely, the survival curve of *Ca* in co-culture with heat-inactivated EcN was indistinguishable from that of the *Ca* monoculture condition (Figure 2B, compare curve with filled squares to that with open triangles). Because we observed a net decrease in relative CFU counting starting at time point 8 h, our data suggest that EcN exerts a fungicidal activity on *Ca* when both species are cultured simultaneously, in vitro, and that this activity requires metabolically active EcN cells.

**Figure 3:**
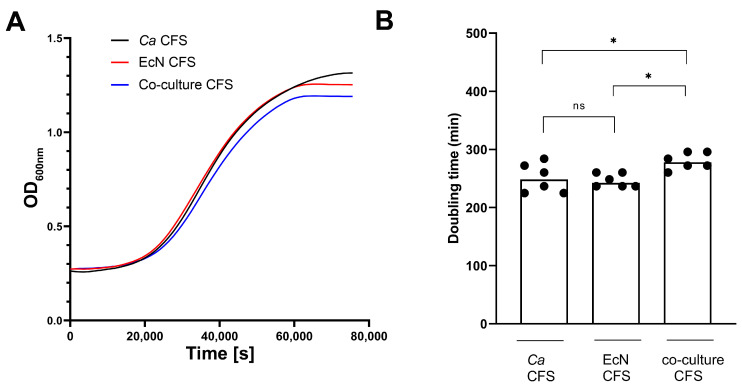
The effect of CFS from EcN monoculture and co-culture assays on *C. albicans* SC5314 growth. (**A**) Representative *Ca* growth curves (OD_600 nm_, *n* = 6) in EcN monoculture (EcN CFS, red curve), *Ca* monoculture (*Ca* CFS, black curve) and co-culture (co-culture CFS, blue curve) CFS supplemented with 2% D-glucose as a function of time. (**B**) Doubling times in minutes are indicated on the *y*-axis for *Ca* SC5314 strain grown in *Ca* monoculture (*Ca* CFS), EcN monoculture (EcN CFS) and in co-culture (co-culture CFS) CFS supplemented with 2% D-glucose (average from *n* = 6 biological replicates). Statistical significance was assigned by performing a one-way ANOVA. *p*-values were subsequently corrected for multiple comparisons using the Bonferroni method (*, *p* < 0.05; ns, not significant).

*Cell-free co-culture supernatant exerts a mild growth-inhibitory activity on C. albicans.* In order to determine if an EcN-released compound is responsible for *Ca* growth inhibition, *Ca* growth rate was quantified during cultivation in cell-free supernatants (CFSs) collected from *Ca* monoculture (control), EcN monoculture and co-culture experiments (Figure 3). CFSs were sterile-filtered, PBS-buffered (at pH 7.4), then supplemented with 2% D-glucose to correct for glucose consumption (See Material and Methods). We first examined *Ca* growth curves under the three different growth conditions (Figure 3A). We noticed that the *Ca* growth curve in co-cultured CFS displayed a mild, but clear, deviation from the growth curves in EcN CFS and *Ca* CFS (Figure 3A, compare blue curve to black and red curves). To robustly quantify this difference, we measured *Ca* doubling times six times independently in each of the three growth conditions (Figure 3B). We confirm a mild, but reproducible, doubling-time increase in *Ca* grown in co-culture CFS, relative to doubling times in EcN and SC5314 monoculture CFS (Figure 3B).

To further investigate the molecular nature of the compound that potentially mediates *Ca* growth inhibition, we applied the CFS from the *Ca*-EcN co-culture and EcN monoculture to Amicon centrifugal filter devices, allowing us to concentrate molecules from dilute samples above a Nominal Molecular Weight Limit (NMWL, see Section 2). We performed an initial experiment using Amicon Ultra-15 3K and Amicon Ultra-15 10K filter devices, with NMWLs of 3 kDa and 10 kDa, respectively. We measured the growth of *Ca* in the resulting concentrated samples by plotting *Ca* growth curves and CFU counts (Figure S1, left panels and right panels, respectively, in A and B). We found that both 3K (Figure S1A) and 10K (Figure S1B) concentrates from the *Ca*-EcN co-culture CFS were still capable of exerting growth-inhibitory activity on *Ca*, suggesting the possible implication of a compound with a molecular weight higher than 10 kDa.

Taken together, our results suggest that *Ca* growth inhibition by EcN can be mildly mediated by a CFS component, but only when both species are cultivated simultaneously.

**Figure 4:**
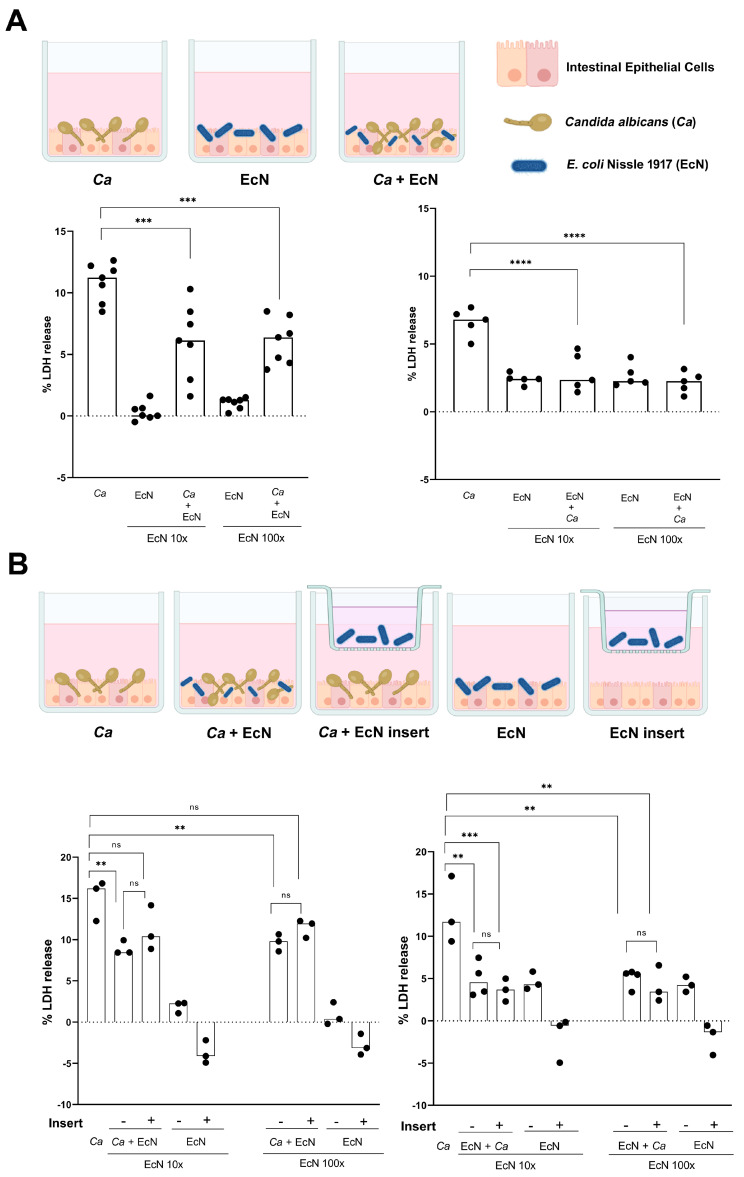
EcN treatment decreases *C. albicans* SC5314−mediated intestinal cell damage. (**A**) Experimental design of intestinal epithelial cell infection model. The images were generated using BioRender (https://www.biorender.com/, 3 December 2022). Bottom left panel, intestinal epithelial cells were infected with *Ca* (at MOI 10), and after 6 h of incubation at 37 °C cells were treated with EcN (10× and 100× relative to *Ca*). Bottom right panel, cells were pre-treated first with EcN (10× and 100×) and, 6 h later, infected with *Ca* (at MOI 10). After 24 h of total incubation in both conditions, the LDH release in the supernatant was measured as described in the Section 2. Medians from 5–7 independent experiments were plotted. Statistical significance was calculated using a one-way ANOVA. *p*-values were subsequently corrected for multiple comparisons using the Bonferroni method (***, *p* < 0.001; ****, *p* < 0.0001). (**B**) Experimental design of intestinal epithelial-cell infection model using transwell cell culture inserts. Bottom left panel, intestinal epithelial cells were infected first with *Ca* (at MOI 10) and, 6 h later, treated with EcN (10× and 100×) that was added either separately in the insert (+) or in direct contact with enterocytes (−) and Ca. Bottom right panel, intestinal epithelial cells were pre-treated first with EcN (10× and 100×) that was added either separately in the insert (+) or in direct contact with enterocytes (−), and 6 h later, enterocytes were infected with *Ca* (at MOI 10). After 24 h of total incubation in both conditions, the percent LDH release (% LDH release, *y*-axis) in the supernatant was measured. Medians from 3–4 independent experiments were plotted. Statistical significance was calculated using a two-way ANOVA. *P*-values were subsequently corrected for multiple comparisons using the Bonferroni method (**, *p* < 0.01; ***, *p* < 0.001; ns, not significant).

*EcN protects intestinal epithelial cells from C. albicans-mediated damage.* Our findings that EcN exerts growth-inhibitory activity against *Ca* when both species are interacting together led us to test whether EcN is capable of inhibiting *C. albicans*’ ability to cause damage to intestinal cells. We used a relevant human intestinal epithelial cell model composed of brush border-forming C2BBe1 cells and mucus-secreting HT29-MTX cells at a 70:30 ratio, respectively [24]. This model was shown as a versatile and suitable in vitro model of the human intestinal epithelium [27]. We infected intestinal epithelial cells with *Ca* at an MOI of 10 for 6 h, then added EcN at ratios of 10× and 100× relative to *Ca* for 24 h (Figure 4A, left panel). The potential for *Ca* necrotic cell damage, as determined by the concentration of epithelial cytosolic lactate dehydrogenase (LDH) in the culture supernatants, was quantified five to seven times independently (Figure 4A). Infection with *Ca* alone caused significant damage to enterocytes, reflected in LDH release reaching levels as high as 11.2% (relative to a Triton-induced high control corrected for spontaneous cell death of uninfected enterocytes, as described in the Section 2) (Figure 4A, left panel). The addition of 10× and 100× EcN decreased *Ca*-mediated cell damage by ~50% (Figure 4A, left panel). As a control, EcN alone at ratios of 10× and 100× relative to *Ca* caused very low levels of LDH release (Figure 4A, left panel). These results suggest that EcN reduces *Ca*’s ability to damage intestinal cells when administered post-*Ca* infection. We also tested the impact of the addition of EcN 6 h prior to *Ca* infection (i.e., protective effect, Figure 4A, right panel). Infection with *Ca* alone caused less damage to enterocytes (%LDH release = 6.8%) because the exposure time to *Ca* in this condition was 18 h, rather than 24 h (Figure 4A, right panel). We found that the addition of EcN at ratios of 10× or 100× relative to *Ca* totally protected enterocytes from *Ca*-mediated damage, reflected in LDH release levels similar to those observed in the control with EcN alone (Figure 4A, right panel).

Our finding that an EcN-*Ca* co-culture CFS component can be involved in *Ca* growth inhibition led us to investigate whether EcN is able to protect enterocytes from *Ca*-mediated cell damage either (i) distantly (e.g., via the secretion of metabolites/small molecules) or (ii) in physical contact with *Ca* and intestinal cells (Figure 4B). We used a hanging transwell cell culture insert with a pore size of 0.4 µm to separate EcN from the *Ca* and enterocytes (Figure 4B, see schematic representation in upper panel and Section 2). We infected enterocytes with *Ca* (MOI 10), then added EcN 6 h later at ratios of 10× and 100× relative to *Ca*, either separately in the insert or in direct contact with the *Ca* and enterocytes (Figure 4B, left panel). While the direct addition of EcN to *Ca* cells infecting enterocytes decreased *Ca*-mediated cell damage by ~50% (Figure 4B, left panel), the addition of EcN to inserts did not significantly decrease *Ca*-mediated cell damage (Figure 4B, left panel). We also tested the ability of EcN to protect enterocytes from *Ca*-mediated damage, first by adding EcN either directly to enterocytes or distantly in inserts, then infecting enterocytes with *Ca* (Figure 4B, right panel). We found that EcN abolished *Ca*-mediated cell damage both distantly (i.e., EcN added to inserts) and in direct contact with the *Ca* and enterocytes (Figure 4B, right panel). Our results suggest that EcN is capable of protecting epithelial intestinal cells from *Ca*-mediated damage. This protection is achieved both distantly and in direct contact with *Ca* and enterocytes.

**Figure 5:**
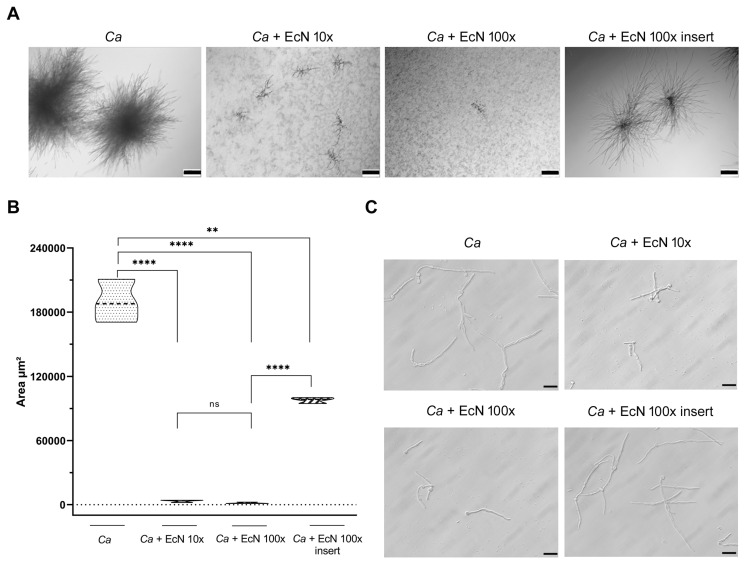
The impact of EcN on *C. albicans* SC5314 microcolony formation. (**A**) Microcolony assay of *Ca* co-incubated with 10× and 100× EcN in RPMI at 37 °C, 5% CO_2_ for 24 h. EcN was added to *Ca* either directly into the well or separately into a transwell cell culture insert. *C. albicans* incubated alone served as a control. After 24 h of incubation, the microcolonies were observed using Zeiss Axio Vert A1 microscope at 5× magnification. Scale bars indicate 200 µm. (**B**) Microcolony area measurements per square micrometer were performed using ImageJ Fiji software v 2.10. *p*-values were calculated using a two-tailed Student’s *t* test (ns, not significant; **, *p* < 0.01; ****, *p* < 0.0001). (**C**) Filamentation was induced under the same experimental conditions as those for microcolony formation. *Ca* cells were co-incubated directly with 10× and 100× EcN cells or distantly through the use of transwell inserts that separated both species. *Ca* hyphal formation was evaluated after 8 h of incubation. Hyphae were observed with Zeiss Axio Vert A1 microscope at 40× magnification. Scale bars indicate 20 µm.

*EcN alters C. albicans microcolony formation*. *Ca* can form pathogenic microcolonies on epithelial cells to invade tissues [11]. We reasoned that EcN’s ability to protect intestinal epithelial cells from *Ca*-mediated damage could be exerted, at least in part, through the inhibition of *C. albicans* microcolony formation. We incubated *Ca* cells in RPMI medium at 37 °C with 5% CO_2_ for 24 h. Under these conditions, *Ca* formed highly dense microcolonies (Figure 5A, left panel, *Ca*). The presence of EcN cells in contact with *Ca*, at either 10:1 or 100:1 ratios, strongly reduced microcolony formation (Figure 5A, panels *Ca* + EcN 10× and *Ca* + EcN 100×). We further tested whether EcN is capable of distantly (e.g., through the release of soluble components) inhibiting microcolony formation by adding it to hanging transwell cell culture inserts (Figure 5A, right panel). We found that EcN reduced the density of microcolonies formed by *Ca* when added to transwell inserts (Figure 5A, right panel). The assay was repeated three times independently, and the phenotypes were quantified by measuring the microcolony area per square micrometer in each condition (Figure 5B). A drastic inhibition of microcolony size was observed when *Ca* was directly exposed to EcN at ratios of 10:1 or 100:1 (EcN:*Ca*, Figure 5B). Microcolony size was reduced by ~50% when EcN was added to inserts (Figure 5B).

Because hyphal formation is one of the initial drivers of the ability of *Ca* to form microcolonies [11], we tested the impact of EcN on *Ca*’s filamentous growth when it is co-incubated directly or distantly in transwell inserts with *Ca* (Figure 5C). *Ca* formed long and branched hyphae under conditions conducive to microcolony formation (Figure 5C, *Ca* panel). Direct co-incubation of *Ca* with EcN resulted in shorter hyphae (Figure 5C, panels *Ca* + EcN 10× and *Ca* + EcN 100×). Conversely, the addition of EcN to the transwell insert did not alter *Ca*’s filament length (Figure 5C, panel *Ca* + EcN 100× insert), indicating that EcN distantly reduces microcolony formation through a different mechanism.

Taken together, our data indicate that EcN is capable of altering—both distantly and through physical contact—the ability of *Ca* to form microcolonies, a virulence trait that is expressed by *Ca* to invade host tissues.

## 4. Discussion

In this report, we show that EcN antagonizes *Ca* growth when both species directly interact with each other. Our finding that the survival of *Ca* decreases following direct exposure to EcN suggests the implication of a fungicidal factor (Figure 2). On the other hand, CFS from the EcN-*Ca* co-incubation assay—but not from the EcN monoculture—exerted mild growth-inhibitory activity on *Ca* (Figure 3). Taken together, our results suggest that a combination of two mechanisms is likely to be involved in the ability of EcN to inhibit *Ca* growth: (i) a mechanism involving direct contact between EcN and *Ca*, probably with fungicidal activity (Figure 2) and (ii) a mechanism with less potent antifungal activity, whereby a soluble component is likely to be released when EcN is co-incubated directly (Figure 3) or distantly in transwell inserts with *Ca* (Figure 5A). The ability of EcN to potently and mildly inhibit microcolony formation when it interacts directly and distantly with *Ca*, respectively, further supports our hypothesis (Figure 5A). We exploited the *Ca* growth-antagonizing potential of EcN to test the ability of EcN to prevent (i.e., pre-*Candida* infection) or decrease (post-*Candida* infection) *Ca*-mediated intestinal cell damage (Figure 4). We show that EcN is capable of totally preventing *Ca* from causing damage to human intestinal epithelial cells when administered prior to *Ca* infection (i.e., prophylactic effect, Figure 4, right panels). Interestingly, this cell-damage-preventive potential was exerted by EcN when it was co-incubated either distantly or directly with *C. albicans* and enterocytes (Figure 4). When administered post-*Ca* infection, EcN reduced *Ca*-mediated cell damage by 50% (Figure 4A, left panel). Therefore, in the context of our investigation, EcN is more potent at exerting a prophylactic rather than a therapeutic effect.

Bacteria and fungi competitively thrive within ecological niches in a variety of ways, communicating through the secretion of small molecules and metabolites, physical interactions and alterations in the composition and function of the environment [28]. The interaction between *Ca* and *E. coli* was investigated to some extent by other research groups, and it is not necessarily antagonistic. For example, *Ca* can form dual species biofilms with *E. coli*, reflecting a stimulatory rather than an inhibitory interaction [29]. Cabral et al. showed that *E. coli* commensal strain MG1655, a derivative of reference lab strain K-12, outcompetes and kills *Ca* in vitro [21]. Unlike EcN, MG1655 probably exerts its growth-inhibitory activity through the release of a potent fungicidal soluble factor whose activity depends on magnesium availability [21]. We tested whether EcN co-culture CFS inhibitory activity on *Ca* was dependent on magnesium and other metal availability, and found that it was not the case (Figure S2). This suggests that EcN and MG1655 exert their anti-*Candida* activity through different mechanisms. We can speculate that EcN mediates the fungicidal effect by directly interacting with *Ca* cells through a contact-mediated killing mechanism similar to the one occurring between *P. aeruginosa* and *Ca* [30]. The mild growth-inhibitory activity exerted by EcN-*Ca* co-culture CFS on *C. albicans* could be due to the stimulation of the release of a soluble factor following the interaction of *Ca* with EcN, either distantly, through signaling molecules, or directly, through physical contact. A recent investigation on postbiotics produced by EcN in functional yogurt indicated that it exerted moderate antifungal activity on a set of five *Candida* species, including *Ca* [31]. The identity of the molecular component responsible for *Ca* growth inhibition by EcN and the underlying mechanism of action await further investigation.

Previous work has documented the protective effect of bacterial probiotic strains on enterocytes from *Ca*-mediated damage. Using the same intestinal epithelial cell model as the one used here, composed of brush border-forming cells and mucus-secreting cells, Graf et al. showed that the probiotic strain *Lactobacillus rhamnosus* reduced *Ca* translocation through the epithelial barrier and decreased *Ca* hyphal elongation [32]. We show here that EcN protects intestinal epithelial cells against *Ca*-mediated damage (Figure 4) and alters microcolony formation and hyphal development (Figure 5). Damage protection by *L. rhamnosus* is not mediated by a secreted factor and occurs mainly through direct contact with *Ca* and enterocytes [32], whereas EcN mediates protection through both contact-dependent and contact-independent mechanisms (Figure 4). While *L. rhamnosus*’s protective effect was dose-dependent [32], EcN’s protective effect did not further increase when a 10-fold excess of EcN to *Ca* cells was further administered (i.e., from 10× to 100×, Figure 4). It is possible that EcN’s effect on *Ca*-mediated cell damage reached saturation at 10× (Figure 4). In fact, microcolony formation was readily abolished by EcN at 10× (Figure 5A). *L. rhamnosus* and EcN added 6 h after *Ca* infection reduced host cell damage by 60% and 50%, respectively [32] (Figure 4). Strikingly, near 100% protection against *Ca*-mediated enterocyte damage was conferred by EcN when added prior to *Ca* infection (Figure 4). This protection occurred in both contact-dependent and contact-independent fashions, suggesting the implication of a released soluble factor. In addition to a direct effect on *Ca* growth, such a potent prophylactic effect could also be mediated through the stimulation of antimicrobial peptide release by intestinal cells, for example human β-defensin 2 (hBD-2), which carries potent fungicidal activity against *Ca* [33]. Indeed, EcN was shown to stimulate hBD-2 production by Caco-2 cells through the release of flagellin in the culture medium [34]. EcN is also capable of exerting immunomodulatory and anti-inflammatory activities, and has a restorative effect on the epithelial permeability barrier [15]. A study dating back to 1997 demonstrated the ability of orally supplemented EcN to enhance the immune response against systemic *Ca* infection in vivo [35], reflecting the therapeutic potential of EcN even in the context of candidemia.

From an immunological perspective, the in vitro *Ca*– and EcN–enterocyte interaction models similar to those used in our study have been characterized in the literature. Schirbel et al. showed that both yeast and hyphal forms of *Ca* induce the production of hBD-2 as well as the pro-inflammatory cytokines thymic stromal lymphopoietin, IL-6 and IL-8, in Caco-2 cells [36]. They also showed increased expression of immune recognition and chemokine receptors, including TLR2, TLR4 and CXCR1 [36]. *Ca* was able to activate the NF-κB signaling pathway as well as the cFOS gene, which encodes a subunit of the heterodimeric transcription factor AP-1, reflecting an expected induction of a damage response by the infected enterocytes [32]. As stated above, and in contrast to more than 40 different *E. coli* strains tested, EcN was able to strongly induce the expression of hBD-2 in Caco-2 cells [37] and to decrease the inflammasome activation and secretion of IL-18 compared with commensal strain *E. coli* K12 [38]. In a model of Caco-2 cells infected with Crohn’s Disease-associated *E. coli* strain LF82, exposure to EcN decreased the amounts of soluble ICAM1, Gro-α and IL-8 compared with cells infected only with strain LF82 [39]. This points to an anti-inflammatory effect of EcN in the context of prior infection with a gut pathogen through the modification of cytokine expression in enterocytes [39]. Yet, genome-wide expression profiling of uninfected Caco-2 following exposure to EcN, rather, indicated a proinflammatory response, including the specific up-regulation of monocyte chemotactic protein-1 (MCP-1) [40]. Similarly, the impact of EcN CFS on cytokine secretion by mucus-producing cells HT29-MTX (used in this study in combination with Caco-2 clone C2BBe1) was strong, compared, for example, with that of probiotic strain *L. rhamnosus* GG CFS [41]. Relatively high levels of IL-8, MCP-1, TGF-α, TNF-α, granulocyte macrophage colony-stimulating factor and IFN-γ were detected upon exposure of HT29-MTX cells to EcN CFS [41]. EcN CFS also triggered relatively low levels of anti-inflammatory cytokines (IL-4, IL-5 and IL-10) and cytokines with dual inflammatory characteristics (IL-6 and IL-13) [41], indicating that EcN CFSs were more proinflammatory than anti-inflammatory in this context. This somehow contrasts with the beneficial properties of EcN and reflects the complexity of its immunomodulatory properties, which appear to depend on the cellular model used for assessing them.

Historically, the antimicrobial activity of EcN has been investigated mainly against bacterial pathogens. Originally, EcN was used to treat patients for the strong antagonistic activity against closely related enteropathogens [15]. As a commensal to the gastrointestinal tract, EcN should have evolved mechanisms that allow it to efficiently compete with a wide array of species that are part of the intestinal microbiota, including fungal species. EcN typically exerts its direct antibacterial activity against phylogenetically related bacteria through the secretion of two microcins (H47 and M), which are low-molecular-weight peptides displaying potent bactericidal activity [16]. EcN also synthesizes a genotoxin called colibactin, which induces DNA crosslinks, double-strand breaks and chromosome aberrations in eukaryotic cells [42]. Such DNA-alkylating toxins are widespread among bacterial species, and some of them were shown to exert potent growth-inhibitory activity on fungi, including *Ca* and *Aspergillus fumigatus* [43]. Whether colibactin could exert antifungal activity is unknown; in fact, the extent of colibactin’s activity on microbes inhabiting the intestine is still unclear. Hints on the nature of EcN’s antifungal activity could be provided through mining of the metabolomic data available from the literature [18,44,45]. In fact, EcN is phylogenetically closely related to *E. coli* asymptomatic bacteriuria strain 83,972 [46] and the highly virulent uropathogenic strain CFT073 [47]. Only relatively minor genetic variations are found between the three isolates [48], suggesting that they originated from a unique ancestral strain. Interestingly, EcN is more similar to pathogenic CFT073 with respect to genotype and phenotype than the commensal 83,972 strain [48]. Recent studies performed comparative analyses of the metabolomes from the three *E. coli* strains and showed that the metabolome of EcN differs significantly from the others [44,45]. A more recent study examined the metabolomes of EcN and the strain *L. rhamnosus* GG to reveal small molecules with differential growth inhibition of antimicrobial-resistant pathogens [18]. Data from these studies together with those from future investigations could be extensively mined in order to help us better understand the extended therapeutic potential of EcN and how this probiotic strain can exert antifungal activity.

## 5. Conclusions

In this report, we provide evidence that EcN exerts an interesting anti-*Ca* activity to explore for future therapeutic approaches. Probiotics are increasingly used as a treatment for gastrointestinal disorders. They are characterized by their capacity to modulate the immune response, but also to exert antimicrobial activity against many pathobionts of the human gut, including *Ca*. Future work will aim at deciphering the precise molecular mechanisms underlying EcN’s antagonistic activity against *Ca* and assessing its impact, in vivo, in a relevant animal model of *Ca* gut colonization.

## Data Availability

Not applicable.

## Supplementary Materials

The following supporting information can be downloaded at: https://www.mdpi.com/article/10.3390/microorganisms11081929/s1, Figure S1: The effect of Amicon Ultra-15 filter-processed CFS from EcN monoculture and EcN-*Ca* co-culture on *Ca* growth; Figure S2: The addition of magnesium to culture medium does not affect *Ca* growth inhibition by EcN.
