# Supplementary material for: Escherichia coli Nissle 1917 Antagonizes Candida albicans Growth and Protects Intestinal Cells from C. albicans-Mediated Damage
Source: Microorganisms. 2023 Jul 28;11(8):1929. doi: 10.3390/microorganisms11081929 (PMC10457924; doi:10.3390/microorganisms11081929)
Supplement: Supplementary file 1 [file microorganisms-11-01929-s001.zip › microorganisms-2387510-supplementary.pdf]

Escherichia coli Nissle 1917 antagonizes *Candida albicans* growth and protects intestinal cells from *C. albicans*-mediated damage

Supplementary Material

Figure S1

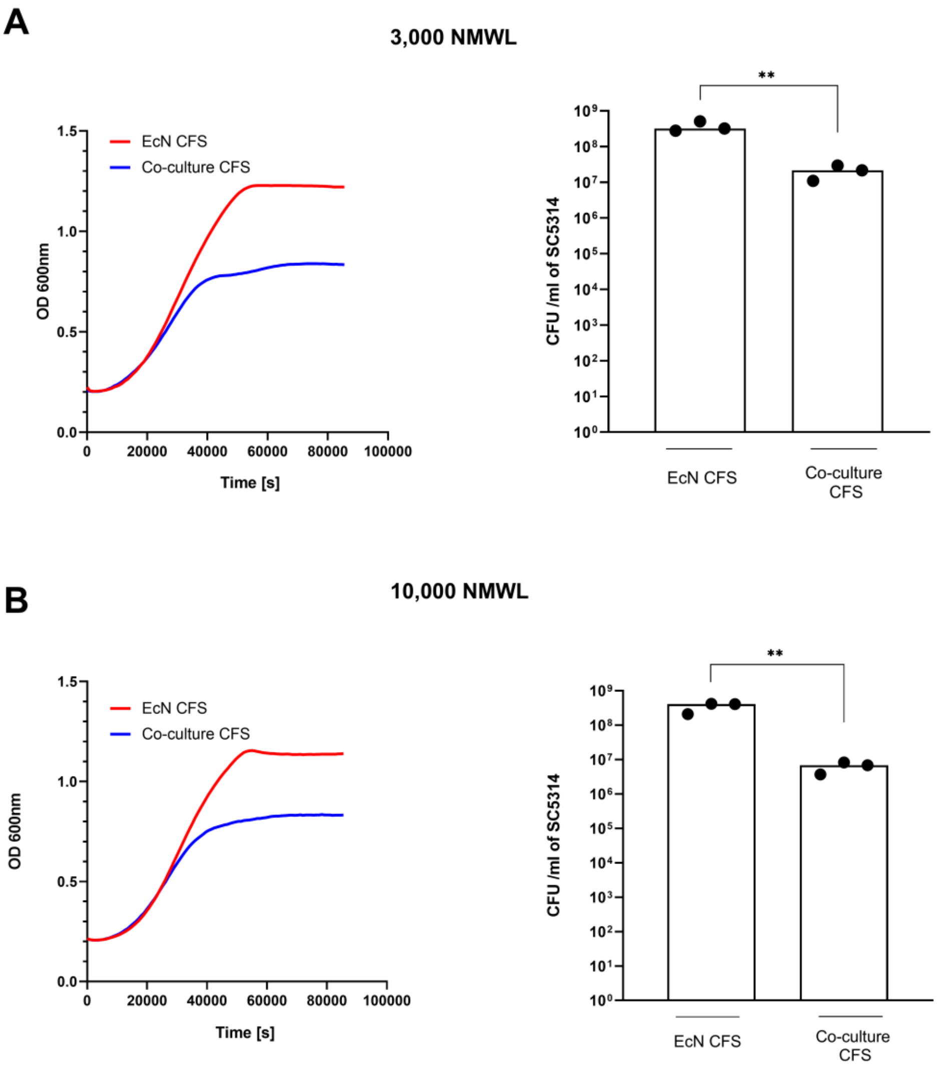

Figure S1. The effect of Amicon Ultra-15 filter-processed CFS from EcN monoculture and EcN-*Ca* co-culture on *Ca*. growth. (A) Left panel, EcN monoculture (EcN CFS, red curve) and EcN-*Ca* co-culture (co-culture CFS, blue curve) CFS were applied to 3,000 NMWL Amicon Ultra-15 filters then used as medium for monitoring growth of *Ca* (OD_{600nm}, *y*-axis, n = 3, one representative curve is shown) as a function of time (seconds, *x*-axis). Right panel, the abundance of *Ca* in EcN monoculture CFS (ECN CFS) and in EcN-*Ca* co-culture CFS (Co-culture CFS) was also determined by counting colony-forming units per milliliter (CFU/mL, *y*-axis) on selective solid agar medium after 24 h of growth. **(B)** Left panel, EcN monoculture (EcN CFS, red curve) and EcN-*Ca* co-culture (co-culture CFS, blue curve) CFS were applied to 10,000 NMWL Amicon Ultra-15 filters then used as medium for monitoring growth of *Ca* (OD_{600nm}, *y*-axis, n = 3) as a function of time (seconds, *x*-axis). Right panel, the abundance of *Ca* in EcN monoculture CFS (EcN CFS) and in EcN-*Ca* co-culture CFS (Co-culture CFS) was also determined by counting colony-forming units per milliliter (CFU/mL, *y*-axis) on selective solid agar medium after 24 h of growth. Statistical analyses were carried out using a two-tailed student *t* test comparing means of *Ca* abundance in EcN CFS versus co-culture CFS (Statistical significance: **, $p<0.01$).

Figure S2

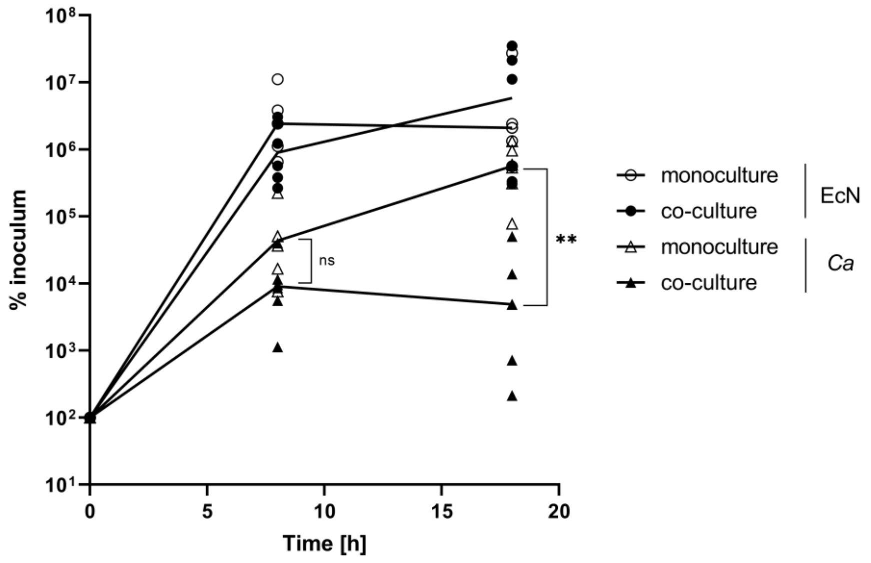

Figure S2. The addition of magnesium to culture medium does not affect *Ca* growth inhibition by EcN. Percent abundance of *Ca* relative to inoculum (% inoculum, *y*-axis, *i.e.* survival) as a function of time (hours, *x*-axis) grown in monoculture supplemented with 200 µM $MgSO_4$ (EcN, open circles; *Ca*, open triangles) and in co-culture with EcN supplemented with 200 µM $MgSO_4$ (EcN, filled circles; *Ca*, filled triangles) was determined by dividing CFU/mL values of each time point (0 h, 8 h and 18 h) by the CFU/mL value of time point 0 and multiplied by 100 (%). Data shown on the *y*-axis represent the mean values of the medians from n=6 independent experiments (*i.e.* performed on different days). Statistical analyses were carried out using a two-tailed Mann-Whitney test comparing monoculture and co-culture % inoculums for each species at time points 8 h and 18 h (ns, not significant ; **, $p < 0.01$).
